# A Wideband Noise and Harmonic Distortion Canceling Low-Noise Amplifier for High-Frequency Ultrasound Transducers

**DOI:** 10.3390/s21248476

**Published:** 2021-12-19

**Authors:** Yuxuan Tang, Yulang Feng, He Hu, Cheng Fang, Hao Deng, Runxi Zhang, Jun Zou, Jinghong Chen

**Affiliations:** 1 Department of Electrical and Computer Engineering, University of Houston, Houston, TX 77004, USA; ytang11@uh.edu (Y.T.); yfeng19@uh.edu (Y.F.); hdeng3@uh.edu (H.D.); 2 Department of Electrical and Computer Engineering, Texas A&M University, College Station, TX 77843, USA; cn_hu_he@hotmail.com (H.H.); fangchengok2007@tamu.edu (C.F.); junzou@ece.tamu.edu (J.Z.); 3 School of Communication and Electronic Engineering, East China Normal University, Shanghai 200241, China; rxzhang@ee.ecnu.edu.cn

**Keywords:** high-frequency ultrasound transducers, low-noise amplifier, noise cancellation, harmonic distortion cancellation, resistive shunt-feedback amplifier, wideband impedance matching

## Abstract

This paper presents a wideband low-noise amplifier (LNA) front-end with noise and distortion cancellation for high-frequency ultrasound transducers. The LNA employs a resistive shunt-feedback structure with a feedforward noise-canceling technique to accomplish both wideband impedance matching and low noise performance. A complementary CMOS topology was also developed to cancel out the second-order harmonic distortion and enhance the amplifier linearity. A high-frequency ultrasound (HFUS) and photoacoustic (PA) imaging front-end, including the proposed LNA and a variable gain amplifier (VGA), was designed and fabricated in a 180 nm CMOS process. At 80 MHz, the front-end achieves an input-referred noise density of 1.36 nV/sqrt (Hz), an input return loss (S_11_) of better than −16 dB, a voltage gain of 37 dB, and a total harmonic distortion (THD) of −55 dBc while dissipating a power of 37 mW, leading to a noise efficiency factor (NEF) of 2.66.

## 1. Introduction

With the recent advancements of high-frequency (>30 MHz) ultrasound transducers [1], such as polyvinylidene fluoride (PVDF) piezoelectric transducers and capacitive micromachined transducers (CMUT), high-frequency ultrasound and photoacoustic imaging have been developed rapidly. The HFUS and PA imaging with improved microscopic resolutions opens many new medical imaging applications [2,3,4,5,6,7] in the fields of ophthalmology, dermatology, photoacoustic microscope, intravascular imaging (IVUS), and systemic sclerosis (SSC).

In HFUS and PA imaging, ultrasound transducers are utilized to detect the acoustic pressure transients and to generate electrical signals accordingly. The electrical signals are then processed by the ultrasonic imaging receiver, in which the low-noise amplifier (LNA) is the key component. To support HFUS and PA imaging, the LNA needs to achieve low noise and high bandwidth simultaneously. Conventionally, the LNA is designed as either a charge-sensitive amplifier (CSA) or a voltage amplifier. Although the CSA provides low noise performance [8,9], the feedback loop formed by the bleeding resistor and the feedback capacitor significantly limits the achievable amplifier bandwidth. The voltage-mode amplifier, such as the resistive shunt-feedback amplifier in [10], achieves large bandwidth and wideband impedance matching, however, the noise performance is poor due to the fixed transconductance of the input transistor for the impedance matching. To improve the noise performance of voltage-mode amplifiers, noise-canceling (NC) techniques [11,12,13] have been recently explored. Reference [12] proposes an LNA utilizing a combination of a common-gate (CG) amplifier and a common-source (CS) amplifier for noise cancellation. Nonetheless, the pseudo-differential structure is prone to gain a mismatch between the CG and CS gain stages. Based on a resistive shunt-feedback amplifier structure, our recent work [13] also demonstrates a noise-canceling wideband LNA, where a common-source auxiliary amplifier is employed to generate an in-phase signal and an out-of-phase noise, with respect to those of the main amplifier, for noise cancellation.

As the outputs of ultrasound transducers are normally in single-ended mode, the LNAs are typically limited to single-ended structures, thus suffering from even-order nonlinear distortion. To suppress the even-order harmonics, single-ended-to-differential conversion circuits have been utilized but with the penalties of additional noise and power consumption. To address this issue, a bulky LC resonator is used as a band-pass filter in [14] to reject the second-order harmonic distortion. In [15], a constant transconductance structure is developed to achieve second-order intermodulation distortion (IM_2_) cancellation. The constant transconductance, however, relies on precisely matched triode transistors.

Power consumption is also a major concern in designing ultrasound LNAs. In beamforming applications where multiple channels are integrated on a single chip, the heat generated by the LNAs can significantly affect chip reliability. Low power consumption is also indispensable in portable ultrasound systems. To reduce LNA power consumption, a step-up balun with two secondary turns is developed in [16] to alleviate the transconductance requirement for impedance matching. The use of balun, however, is not applicable to wideband ultrasound LNAs because of its large area penalty.

In this article, which is an extension of [13,17], a complementary resistive shunt-feedback LNA with both noise and distortion cancellations is presented to simultaneously achieve wideband impedance matching, low noise, and high linearity for HFUS and PA imaging applications. An electrical model of the PVDF ultrasound transducer is also developed to guide the LNA design process. Designed in a 180 nm CMOS technology, the LNA achieves a 0.8 nV/sqrt (Hz) input-referred voltage noise density, a 19 dB voltage gain, and a −59.3 dBc total harmonic distortion (THD) at 80 MHz. An ultrasound front-end with the proposed LNA and a pseudo-differential variable gain amplifier (VGA) is also designed and fabricated. At 80 MHz, measurements show that the front-end achieves an input-referred voltage noise density of 1.36 nV/sqrt (Hz), a better than −16 dB input return loss (S_11_), a voltage gain of 37 dB, and a THD of −55 dBc. The front-end consumes 37 mW of power from a 1.8 V supply and achieves a noise efficiency factor (NEF) of 2.66.

The overall LNA design was carried out by the following procedures. First, the typical electrical parameters of the PVDF ultrasound transducer for HFUS and PA imaging applications were obtained through experimental measurements. The parameters included the resonance frequency, output impedance, and output amplitude of the transducer. Then, an electrical model of the PVDF transducer was developed to facilitate the co-simulation of the LNA with the ultrasound transducer. Second, given the typical PVDF electrical parameters, the LNA design specifications, including gain and bandwidth, input impedance matching, and total harmonic distortion, were defined. Design solutions for improving the bandwidth, noise, linearity, and power efficiency of the LNA were then developed. A test chip was designed and fabricated in a 180 nm CMOS technology, and experimental measurements were carried out to validate the proposed LNA design solutions.

The remainder of this paper is organized as follows. Section 2 describes the model development of the PVDF transducer. Section 3 presents the LNA design solutions for improving bandwidth, noise, linearity, and power efficiency. Section 4 describes the proposed LNA design in a 180 nm CMOS technology. Section 5 presents the chip measurement results and Section 6 concludes the paper.

## 2. PVDF Ultrasound Transducer Model

**Figure 1:**
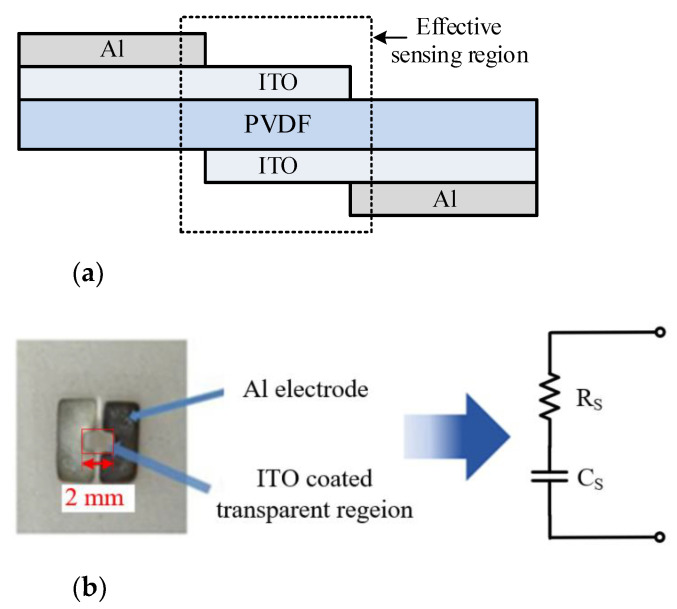
(**a**) Schematic diagram of the PVDF transducer; (**b**) photograph of the PVDF transducer and its electrical model.

**Figure 2:**
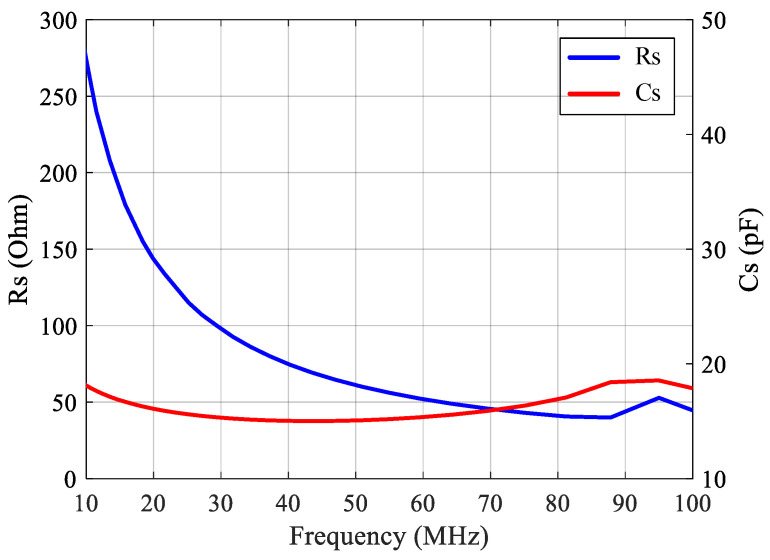
Measured output impedance of the PVDF transducer.

Figure 1a depicts the schematic of the ultrasound transducer, which was made of a 9-μm-thick piezoelectric material PVDF film, whose resonance frequency and bandwidth were around 50~80 MHz and 75~140 MHz, respectively [18,19,20]. The materials of the transducer electrodes were Indium Tin Oxide (ITO) and Aluminum (Al). The fabrication process was conducted as follows: (1) A 9-μm-thick polarized PVDF film was cut into pieces of suitable sizes; (2) With RF sputtering, the 200-nm-thick ITO electrodes were formed on the two surfaces of the PVDF film; (3) With DC sputtering, the aluminum electrodes were formed on the surfaces of the PVDF film. The effective sensing region was the 2 × 2 mm^2^ transparent area at the center. Figure 1b shows the photograph of the PVDF transducer and its electrical model. Measured with an impedance analyzer, Figure 2 presents the electrical output impedance of the transducer, where the series resistance $R_{s}$ was about 50 Ω from 50 MHz to 100 MHz and the series capacitance $C_{s}$ was close to a constant of 16 pF.

**Figure 3:**
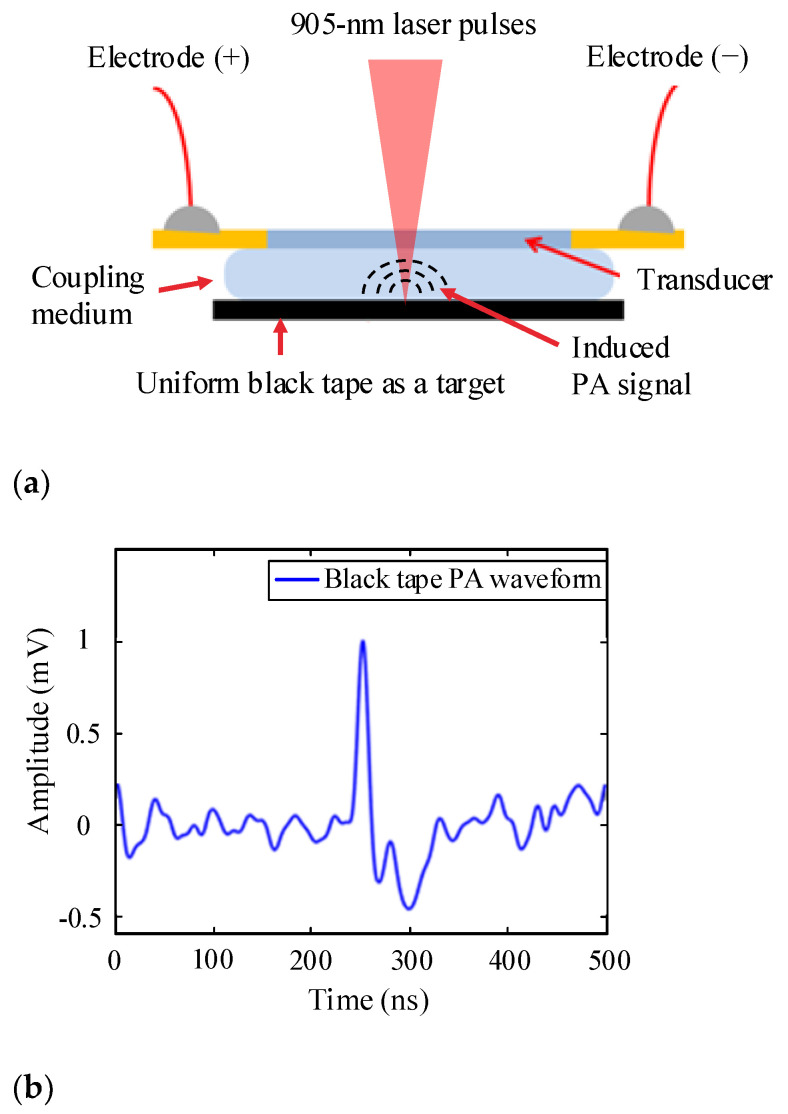
(**a**) Measurement setup for the PA response of the PVDF transducer; (**b**) recorded PA signal induced by laser pulse onto black tape.

As shown in Figure 3a, to measure the photoacoustic response of the transducer, an optically absorptive target made of black tape was used. The target was put under the transducer’s effective sensing region. During the measurement, a small amount of water was added between the transducer and the target surface to improve the acoustic signal coupling efficiency. The 905-nm laser pulses (pulse width: 8 ns, pulse energy: 150 nJ/pulse, repetition rate: 1 kHz) were shot through the transducer to the target. The PA signal excited from the surface of the target was detected by the transducer. The representative PA signal recorded by the transducer is depicted in Figure 3b.

Given that the typical resonance frequency of the PVDF transducer was about 50~80 MHz where the corresponding $R_{s}$, as shown in Figure 2, was about 50 ± 10 Ω and that the typical transducer output signal was 1 mV, as shown in Figure 3b, the LNA along with the post amplifier was thus designed to aim to achieve about 40 dB voltage gain, −50 dBc THD under 1 mV input, and a better than −15 dB input return loss across the transducer resonance frequency range.

## 3. LNA Design and Analysis

### 3.1. Resistive Shunt-Feedback

**Figure 4:**
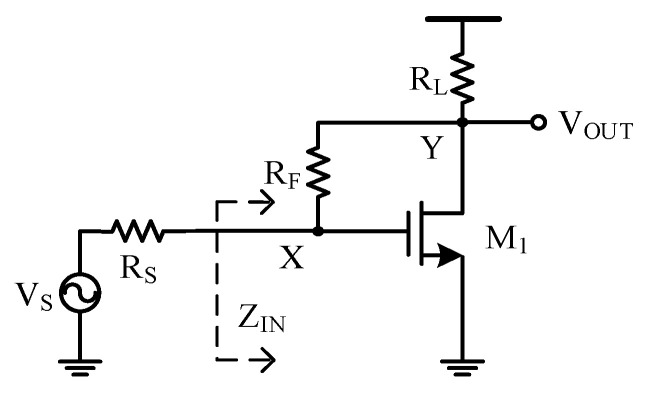
Resistive shunt-feedback amplifier with high bandwidth and wideband impedance matching [13].

The resistive shunt-feedback amplifier, as depicted in Figure 4, was chosen as the basic building block for the proposed high-frequency LNA. The resistive shunt-feedback structure simultaneously offered a high *f*_−3dB_ bandwidth and wideband impedance matching.

Neglecting the effect of parasitic capacitance $C_{gs}$ of the input transistor *M*_1_ and assuming $R_{L}\;\gg {\;R}_{F}$, the input impedance of the shunt-feedback amplifier could be derived as

(1) $$Z_{IN}=\frac{R_{F}{\;+\;R}_{L}}{{1\;+\;g}_{m1}R_{L}}\;\approx \;\frac{1}{g_{m1}},\;$$

where $g_{m1}$ is the transconductance of transistor *M*_1_. The input impedance matching condition was then obtained as

(2) $$R_{S}{=Z}_{IN}=\frac{1}{g_{m1}},$$

where $R_{S}$ is the source impedance.

Next, we derived the signal gain of the amplifier $\frac{V_{Y,S}}{V_{S}}$. The gain from node *X* to node *Y* was derived as

(3) $$\frac{V_{Y,S}}{V_{X,S}}=\frac{\left(1-g_{m1}R_{F}\right)R_{L}}{R_{F}{+R}_{L}}\;\approx \;1-g_{m1}R_{F},\;$$

where $V_{Y,S}$ and $V_{X,S}$ are the signals at the amplifier output node and the gate node of transistor *M*_1_, respectively. Then, under the input impedance matched condition, the signal gain $\frac{V_{Y,S}}{V_{S}}$ was derived as

(4) $$\frac{V_{Y,S}}{V_{S}}=\frac{V_{Y,S}}{V_{X,S}}\times \frac{V_{X,S}}{V_{S}}=\left(1-g_{m1}R_{F}\right)\frac{R_{S}}{R_{S}{+Z}_{IN}}=\frac{1}{2}\left(1-\frac{R_{F}}{R_{S}}\right).$$

The major noise components of the amplifier were the thermal noises of $R_{S}$ and the amplifier input transistor *M*_1_. The noise factor of the amplifier [11] could be derived as

(5) $$F\;>\;1+\frac{{4kT\gamma \;\times \;Z}_{IN}}{{4kTR}_{S}}+\chi =1+\gamma +\chi >2,.$$

where k is the Boltzmann’s constant, T is the temperature in Kelvins, γ is the channel thermal noise coefficient and 1 < γ < 2 for submicron n-channel MOSFETs [21], and χ represents the flicker noise and the thermal noise induced by other parts of the circuit (e.g., $R_{F}$ and $R_{L}$). From Equation (5), the achievable noise figure (NF) was larger than 3 dB and was often larger than 5 dB practically. The noise performance of the wideband resistive shunt-feedback amplifier clearly needs to be improved.

### 3.2. Feedforward Noise Cancellation

**Figure 5:**
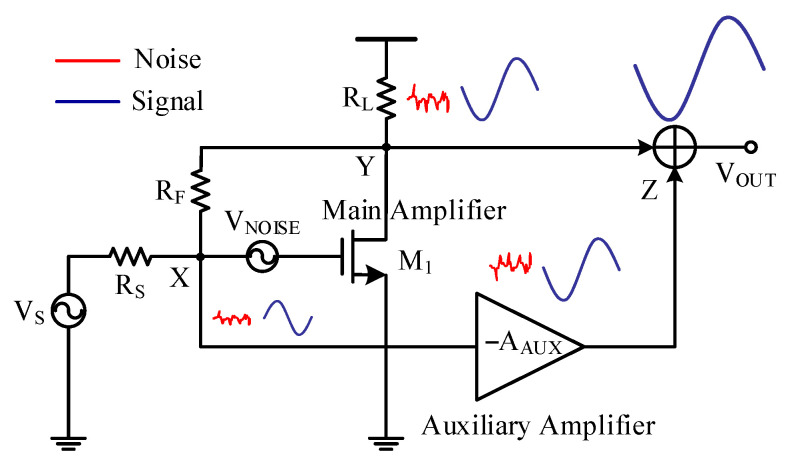
Block diagram of the feedforward noise-canceling technique [13].

To improve the noise performance, feedforward noise cancellation [13] was exploited. Figure 5 depicts the noise-canceling technique where an auxiliary amplifier was used to generate an in-phase signal and an out-of-phase noise with respect to those of the main amplifier *M*_1_. The main amplifier *M*_1_ had an inverting signal gain, as shown in Equation (4). The noise gain of the main amplifier *M*_1_ could be derived as

(6) $$\frac{V_{Y,N}}{V_{X,N}}=1\;+\;\frac{R_{F}}{R_{S}},$$

where *V*_X,N_ denotes the input-referred thermal noise of *M*_1_, and *V*_Y,N_ is the corresponding output noise. From Equation (6), the main amplifier *M*_1_ had a non-inverting gain for noise. To cancel the noise of *M*_1_, the auxiliary amplifier, as shown in Figure 5, was designed to have an inverting amplification for both the signal and the noise at node *X*. With the main amplifier and the auxiliary amplifier exhibiting opposite noise gains, noise cancellation can thus be achieved.

**Figure 6:**
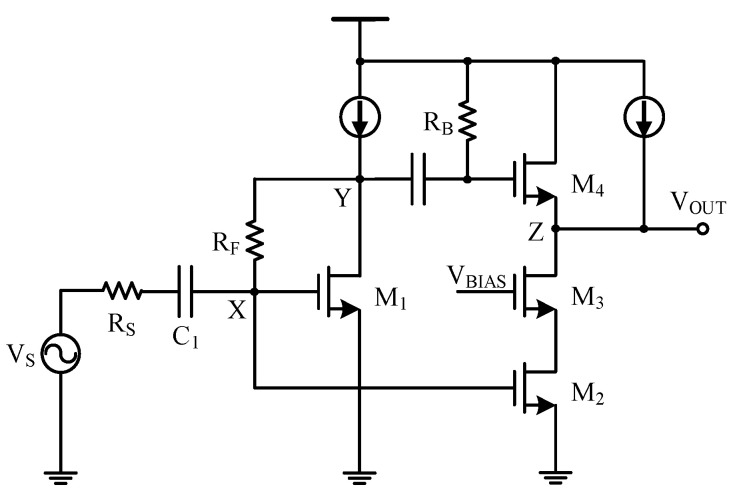
Noise-canceling resistive shunt-feedback LNA [13].

Figure 6 shows the transistor-level implementation of the noise-canceling resistive shunt-feedback LNA, where transistor *M*_1_ worked as the main amplifier, *M*_2_ with a cascode structure worked as the auxiliary amplifier, and *M*_4_ worked as a source follower combining the outputs of both amplifiers. The main amplifier and the auxiliary amplifier were connected in parallel with respect to node *X*. The input impedance condition of the LNA could be derived to be the same as in Equation (2).

Ignoring the small gain reduction due to the source follower, the signal gain $\frac{V_{Z,S,M}}{V_{S}}$ and the noise gain $\frac{V_{Z,N,M}}{V_{X,N}}$ at node *Z* contributed by the main amplifier could be obtained as

(7) $$\frac{V_{Z,S,M}}{V_{S}}=\frac{V_{Y,S,M}}{V_{S}}=\frac{1}{2}\left(1-\frac{R_{F}}{R_{S}}\right),$$

and

(8) $$\frac{V_{Z,N,M}}{V_{X,N}}=\frac{V_{Y,N,M}}{V_{X,N}}=1\;+\;\frac{R_{F}}{R_{S}}.$$

The signal gain $\frac{V_{Z,S,A}}{V_{S}}$ and the noise gain $\frac{V_{Z,N,A}}{V_{X,N}}$ of the auxiliary amplifier were derived as

(9) $$\frac{V_{Z,S,A}}{V_{S}}=\frac{V_{Z,S,A}}{V_{X,S,A}}\times \frac{V_{X,S,A}}{V_{S}}=-\frac{1}{2}\frac{g_{m2}}{g_{m4}},$$

and

(10) $$\frac{V_{Z,N,A}}{V_{X,N}}=-\frac{g_{m2}}{g_{m4}},\;$$

where $g_{m2}$ and $g_{m4}$ are the transconductance of transistors *M*_2_ and *M*_4_, respectively.

With Equations (7)–(10), the noise-canceling condition at node *Z* could be obtained as

(11) $$\frac{V_{Z,N,M}}{V_{X,N}}+\frac{V_{Z,N,A}}{V_{X,N}}=\left(1\;+\;\frac{R_{F}}{R_{S}}\right)-\frac{g_{m2}}{g_{m4}}=0,\;\;\;\;\;\;\;\;\;\;\;\;\;\;\;\;\;\;\;\;\;\;\;\;⟹\;1\;+\;\frac{R_{F}}{R_{S}}=\frac{g_{m2}}{g_{m4}},$$

and the total signal gain $A_{S,TOT}$ was given by

(12) $$A_{S,TOT}=\frac{V_{Z,S,M}}{V_{S}}+\frac{V_{Z,S,A}}{V_{S}}=-\frac{R_{F}}{R_{S}}.$$

Under the noise-canceling condition in Equation (11), the noise of the main amplifier *M*_1_ was canceled by the auxiliary amplifier *M*_2_. The input-referred noise of the overall amplifier was only determined by the auxiliary amplifier *M*_2_ and could be made small with a large $g_{m2}$ without impairing the impedance matching condition, which is only determined by $g_{m1}$ as shown in Equation (2). The LNA thus achieved both wideband impedance matching and low noise performance.

### 3.3. Complementary CMOS Topology

**Figure 7:**
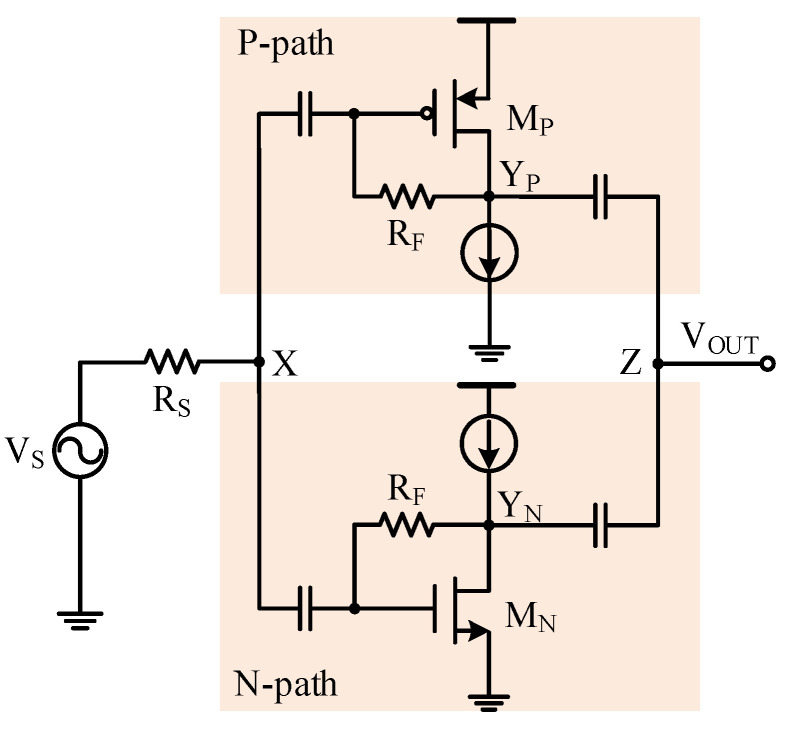
Complementary resistive shunt-feedback amplifier.

To suppress the second-order harmonics in the single-ended ultrasound LNA structure, complementary CMOS topology was investigated [17]. As depicted in Figure 7, the complementary CMOS amplifier consisted of a PMOS-based resistive shunt-feedback amplifier *M*_P_ in parallel with an NMOS-based resistive shunt-feedback amplifier *M*_N_. The drain current in each sub-amplifier as a function of the input signal was respectively obtained as [22]:

(13) $$i_{ds,P}\;{=I}_{DS,P\;}{+\;g}_{m,P}(-v_{gs})\;+\;\frac{1}{2!}g_{m,P}^{\prime }{\left(-v_{gs}\right)}^{2}\;+\;\frac{1}{3!}g_{m,P}^{\prime \prime }{\left(-v_{gs}\right)}^{3}\;+\;\ldots \;,$$

and

(14) $$i_{ds,P}\;{=I}_{DS,N\;}{\;+g}_{m,N}(-v_{gs})\;+\;\frac{1}{2!}g_{m,N}^{\prime }{\left(-v_{gs}\right)}^{2}\;+\;\frac{1}{3!}g_{m,N}^{\prime \prime }{\left(-v_{gs}\right)}^{3}\;+\;\ldots \;,$$

where $g_{m,P}^{\prime }$, $g_{m,N}^{\prime }$, $g_{m,P}^{\prime \prime }$ and $g_{m,N}^{\prime \prime }$ are the first-order and the second-order derivatives of the transconductances $g_{m,P}$ and $g_{m,N}$ with respect to the gate-to-source voltage $v_{gs}$, respectively.

By summing the two currents, the output current of the complementary amplifier was obtained as

(15) $$i_{\mathrm{out}}{=i}_{\mathrm{ds},N}-i_{\mathrm{ds},P}{=(g}_{m,N}{\;+\;g}_{m,P}{)(v}_{\mathrm{gs}})\;+\;\frac{1}{2!}\left(g_{m,N}^{\prime }-g_{m,P}^{\prime }\right){{(v}_{\mathrm{gs}})}^{2}\;+\;\frac{1}{3!}\left(g_{m,N}^{\prime \prime }{\;+\;g}_{m,P}^{\prime \prime }\right){{(v}_{\mathrm{gs}})}^{3}+\ldots .$$

**Figure 8:**
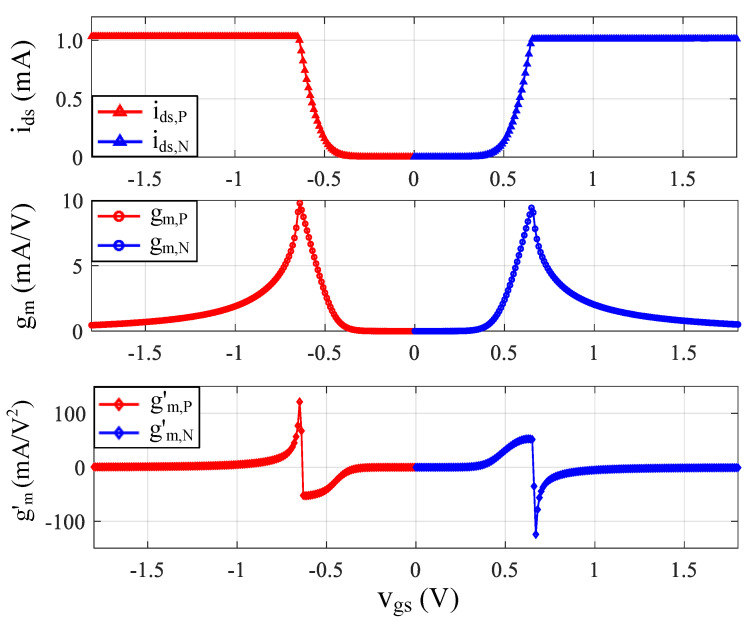
Simulated *i*_ds_, *g*_m_ and *g*’_m_ of a 240-μm/0.18-μm PMOS *M*_P_ and a 120-μm/0.18-μm NMOS *M*_N_ for the complementary resistive shunt-feedback amplifier.

From Equation (15), it can be observed that the second-order harmonic can be largely canceled if $g_{m,P}^{\prime }\;\approx {\;g}_{m,N}^{\prime }$. The simulated drain currents ($i_{\mathrm{ds}}$), the transconductance ($g_{m}),$ and the first-order derivative of the transconductance ($g_{m}^{\prime }$) of a 240-μm/0.18-μm PMOS *M*_P_ and a 120-μm/0.18-μm NMOS *M*_N_ are plotted in Figure 8. With proper biasing, the $g_{m,P}$ and $g_{m,N}$ can be well-defined to achieve $g_{m,P}^{\prime }\;\approx {\;g}_{m,N}^{\prime }$. As the second-order harmonic dominated the nonlinearity of the single-ended LNA, the proposed complementary CMOS amplifier structure provides a low-cost solution to attain good linearity while avoiding the noise and power consumption penalties of a dedicated single-ended-to-differential conversion circuit.

With the complementary CMOS topology, the input impedance matching condition was jointly determined by both the *M*_P_ and *M*_N_ transistors and could be obtained as

(16) $$R_{S}\;{=Z}_{IN}=\frac{1}{g_{m,P}{\;+\;g}_{m,N}}.$$

The signal gain of the complementary resistive shunt-feedback amplifier was derived as

(17) $$\frac{V_{Z,S}}{V_{S}}=\left(\frac{V_{YP,S}}{V_{X,S}}\;+\;\frac{V_{YN,S}}{V_{X,S}}\right)\times \frac{V_{X,S}}{V_{S}}=\left[\left(1-g_{m,P}R_{F}\right)+\left(1-g_{m,N}R_{F}\right)\right]\frac{R_{S}}{R_{S}{+Z}_{IN}}.\;=\frac{1}{2}\left(2-\frac{R_{F}}{R_{S}}\right).\;\;$$

### 3.4. Current-Reuse Technique

**Figure 9:**
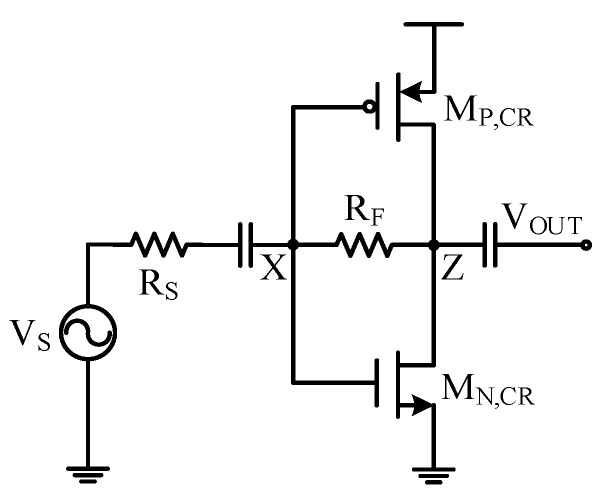
Current-reuse resistive shunt-feedback amplifier.

To reduce the power consumption of the LNA, the current-reuse technique [23] was investigated. Modifying the complementary amplifier in Figure 7 by stacking the P-path amplifier with the N-path amplifier and removing the biasing current sources, the current-reuse resistive shunt-feedback amplifier was constructed and is shown in Figure 9. The input impedance matching condition of the current-reuse amplifier was similar to Equation (16) and was obtained as

(18) $$R_{S}\;{=Z}_{IN}=\frac{R_{F}{+r}_{out,P,CR}{//r}_{out,N,CR}}{1+\left(g_{m,P,CR}{+g}_{m,N,CR}\right){\times \;r}_{out,P,CR}{//r}_{out,N,CR}}\approx \;\frac{1}{g_{m,P,CR}{+g}_{m,N,CR}},\;$$

where $g_{m,P,CR}$ and $g_{m,N,CR}$ are the transconductance of transistors *M*_P,CR_ and *M*_N,CR_, respectively, assuming $r_{out,P,CR}{//r}_{out,N,CR}\;\gg {\;R}_{F}$. Under the input impedance matched condition, the signal gain was derived with the superposition principle as

(19) $$\frac{V_{Z,S}}{V_{S}}=\frac{V_{Z,S}}{V_{X,S}}\times \frac{V_{X,S}}{V_{S}}=\frac{1}{2}\left[\left(1-g_{m,P,CR}R_{F}\right)+\left(1-g_{m,N,CR}R_{F}\right)\right]=\frac{1}{2}\left(2-\frac{R_{F}}{R_{S}}\right),$$

which was the same as Equation (17). In the current-reuse structure, the *M*_P,CR_ and *M*_N,CR_ needed to be sized so that the output common-mode voltage was close to half *V*_DD_. The input common-mode voltage was set by the output common-mode voltage through the feedback resistor $R_{F}$. Compared to the complementary resistive shunt-feedback amplifier in Figure 7, the current-reuse structure only required half of the DC current to maintain the impedance matching condition, thus reducing the amplifier power consumption by almost half. Removing the biasing current sources also allowed a low supply voltage to be used, as long as *M*_P,CR_ and *M*_N,CR_ were in saturation, which helped to further reduce the power consumption.

Simulation results [24] showed that the path stacking of the complementary resistive shunt-feedback amplifier brought a 1.2 mA DC current reduction and the use of a 1.3 V supply further reduced the power consumption by 9 mW, while the LNA could still maintain a *f*_−3dB_ bandwidth of larger than 150 MHz. The use of a low *V*_DD_, however, mandated large sized transistors, especially in the auxiliary amplifiers, to maintain low noise performance. This led to a large parasitic capacitance at the amplifier input node, which could degrade the amplifier S_11_ at high frequencies. In designing the low-*V*_DD_ current-reuse amplifier with feedforward noise-canceling, the *V*_DD_ needs to be properly selected to tradeoff between noise, power saving, bandwidth, and high-frequency S_11_.

## 4. Proposed LNA Design

**Figure 10:**
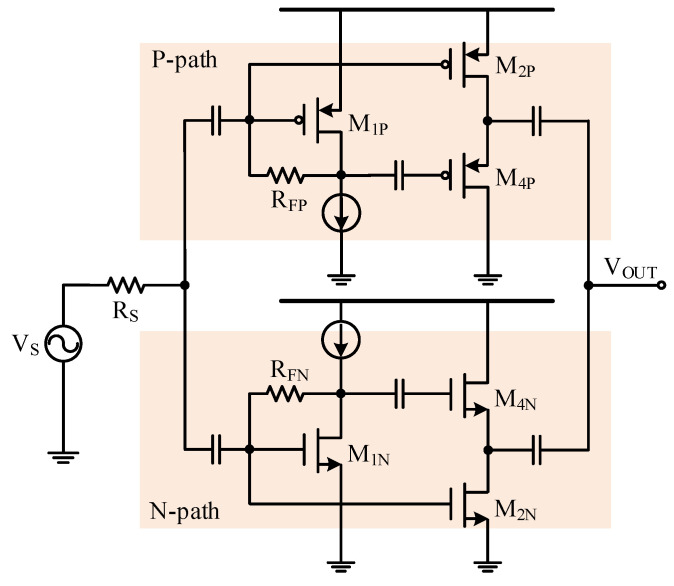
Proposed resistive shunt-feedback LNA with the feedforward noise-canceling technique and the complementary topology [17].

A resistive shunt-feedback LNA with the feedforward noise-canceling technique and the complementary topology was designed in a 180 nm CMOS technology. As shown in Figure 10, the complementary topology was formed of both the N-path and the P-path amplifiers, where each path employed resistive shunt-feedback with feedforward noise cancellation. In the figure, *M*_1P_ and *M*_1N_ are the main amplifiers, *M*_2P_ and *M*_2N_ are the auxiliary amplifiers, and *M*_4P_ and *M*_4N_ are the analog combiners, respectively. The sizes of the *M*_1P_ and *M*_1N_ transistors were 240-μm/0.18-μm and 120-μm/0.18-μm, respectively. Both transistors were biased with 1 mA current. The corresponding $g_{m,1P}$ and $g_{m,1N}$ were 9.81 mA/V and 9.57 mA/V, respectively, leading to an input impedance $Z_{IN}$ of about 51.6 Ω. The corresponding $g_{m,1P}^{\prime }$ was 67 mA/V^2^ and $g_{m,1N}^{\prime }$ was 61 mA/V^2^ and this helped to achieve the cancellation of the second-order harmonic distortion. The size of the *M*_2P_ and *M*_2N_ transistors were designed as 960-μm/0.18-μm and 480-μm/0.18-μm with the corresponding $g_{m,2P}$ being 36.3 mA/V and $g_{m,2N}$ being 38.3 mA/V to achieve low noise performance.

**Figure 11:**
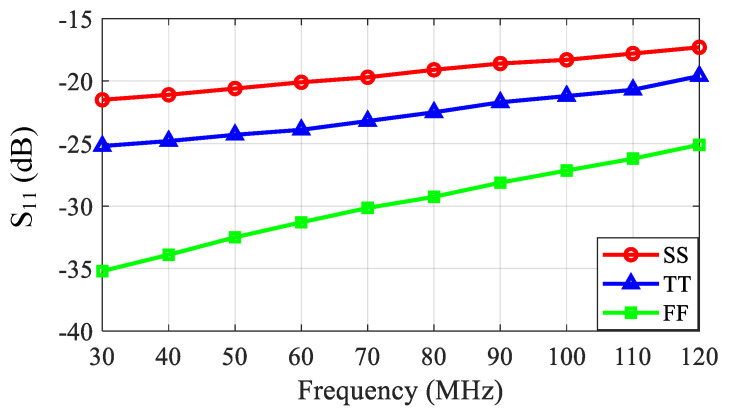
Simulated S_11_ of the proposed LNA, where SS, TT, and FF stand for the slow PMOS and NMOS, typical PMOS and NMOS, and fast PMOS and NMOS process corners, respectively.

**Figure 12:**
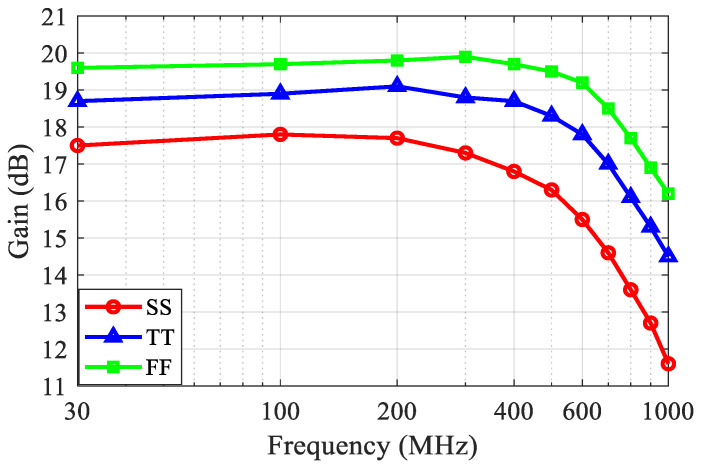
Simulated frequency response of the proposed LNA.

Figure 11 and Figure 12 show the simulated S_11_ and frequency response of the LNA, respectively. The S_11_ was better than −17 dB over the desired ultrasound transducer operation frequency range. The S_11_ at higher frequencies was slightly degraded by the main amplifier input node capacitance. The frequency response shows that the LNA had a gain of 19 dB, up to 120 MHz, in the typical corner and a *f*_−3dB_ bandwidth of 770 MHz. The gain variation over the process, voltage, and temperatures (PVTs) was less than 2 dB.

**Figure 13:**
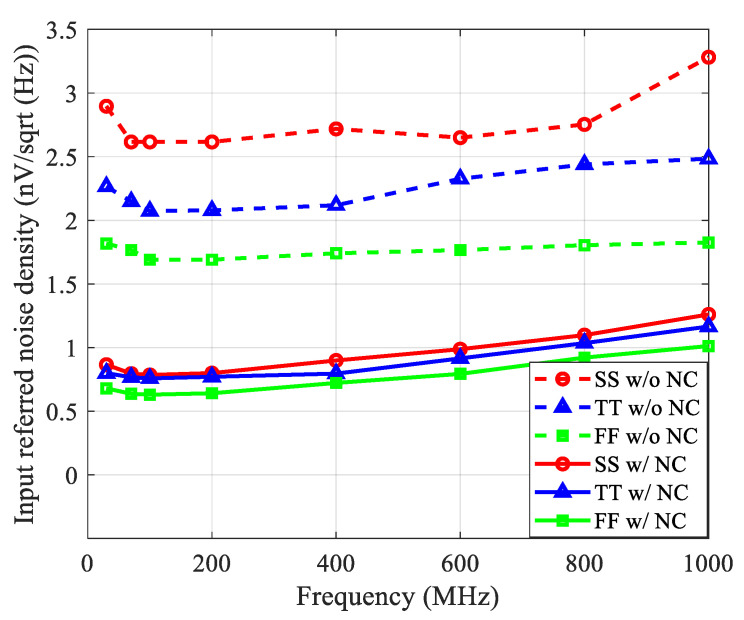
Simulated input-referred voltage noise density of the proposed LNA.

As shown in Figure 13, with feedforward noise cancellation, the amplifier input-referred voltage noise density was reduced by about 3× over PVTs. The input-referred voltage noise density was less than 0.8 nV/sqrt (Hz) over a 30–120 MHz frequency range. The THD simulation was also performed, and the simulation results are summarized in Table 1. The signal generated by the transducer was in the range of 0.5–2 mV peak-to-peak, corresponding to an input signal power of −62 dBm to −50 dBm in a 50 Ω terminated system. As shown in Table 1, the complementary CMOS topology provided a THD improvement of larger than 9 dB across the input signal range.

## 5. Measurement Results and Analysis

**Figure 14:**
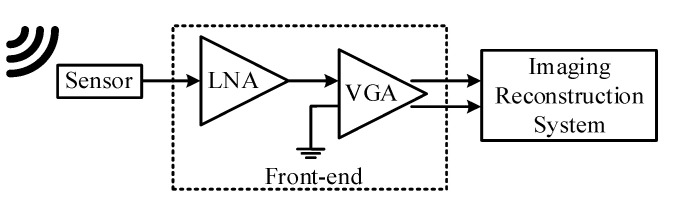
Block diagram of the HFUS and PA imaging front-end.

**Figure 15:**
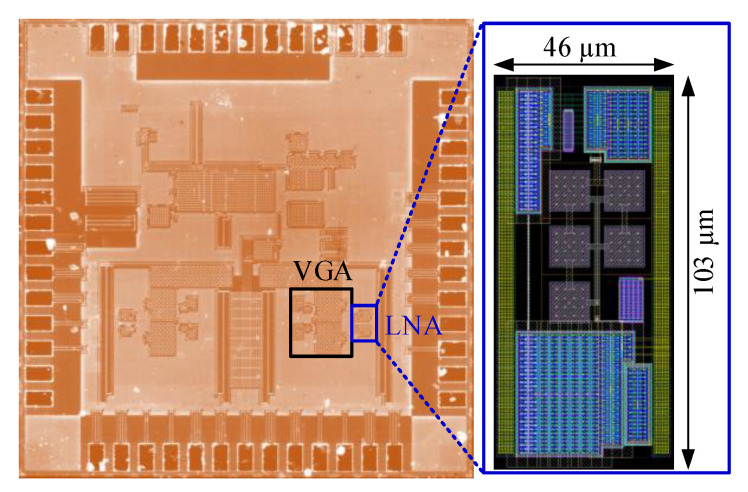
Die photo of the ultrasound front-end.

**Figure 16:**
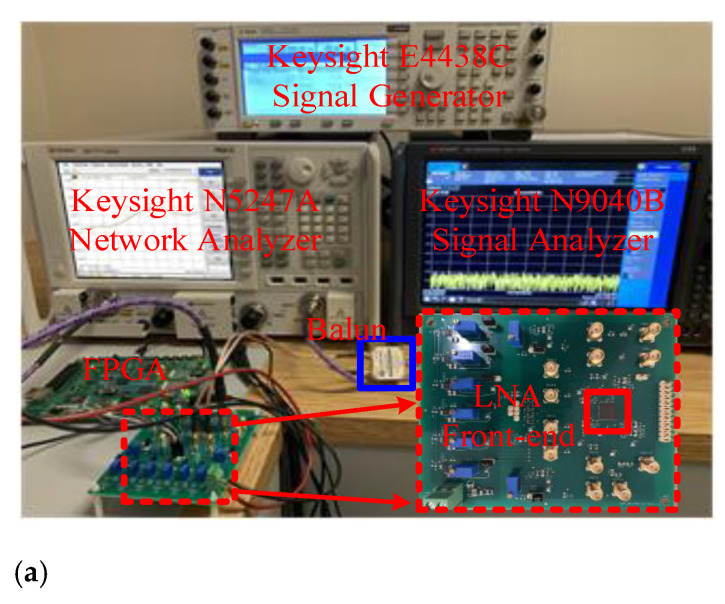

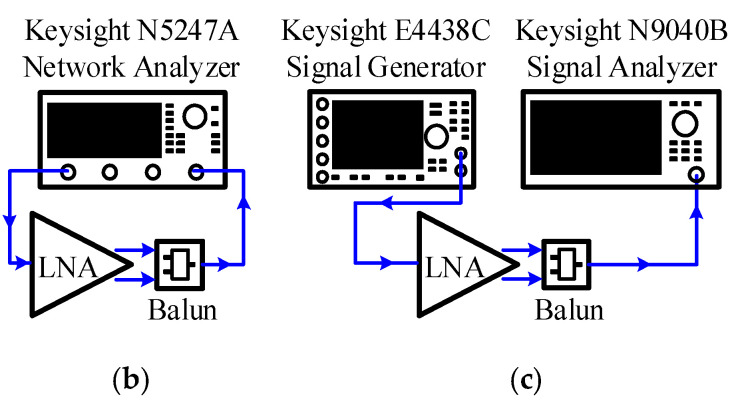
(**a**) Measurements setup; (**b**) S_11_ and frequency response measurement; and (**c**) noise density and THD measurement.

A high-frequency ultrasound and photoacoustic imaging front-end, as shown in Figure 14, including the proposed LNA and a pseudo-differential VGA, has been developed and fabricated in a one-poly six-metal (1P6M) 180 nm bulk CMOS process with a core area of 380 μm × 350 μm. The gain of the VGA is programmable, ranging from 20 to 32 dB with a 6-dB gain step. The Figure 15 shows the die photo of the front-end. Figure 16 shows the measurements setup. The S_11_ and frequency response were measured with the Keysight N5247A network analyzer. The noise density and the THD were measured with the Keysight E4438C signal generator and the Keysight N9040B signal analyzer.

The Gain Method [25] has been applied to measure the NF. The NF in the Gain Method could be expressed as

(20) $${\mathrm{NF}=P}_{\mathrm{NOUTD}}+174\;\mathrm{dBm}/\mathrm{Hz}-A_{V},\;$$

where ${\;P}_{\mathrm{NOUTD}}$ is the measured output voltage noise density, 174 dBm/Hz is the noise density of 290°K ambient noise, and $A_{V}$ is the measured front-end gain. Based on the measured NF, the corresponding input-referred voltage noise density, $e_{\mathrm{NI}}$, in a 50 Ω terminated system was obtained as

(21) $$e_{\mathrm{NI}}=\sqrt{{4kT\;\times \;R}_{50}\times \left[{\left(10^{NF/10}\right)}^{2}-1\right]}.\;$$

**Figure 17:**
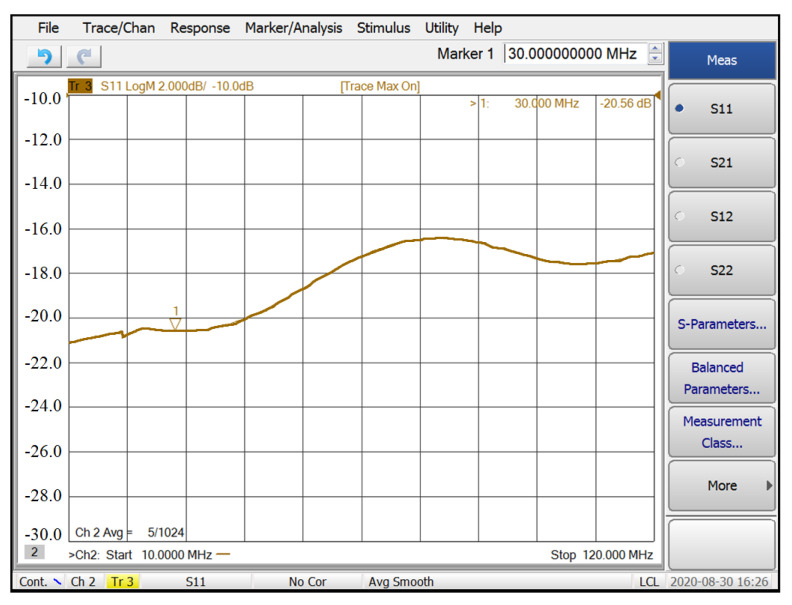
The measured S_11_ of the front-end.

**Figure 18:**
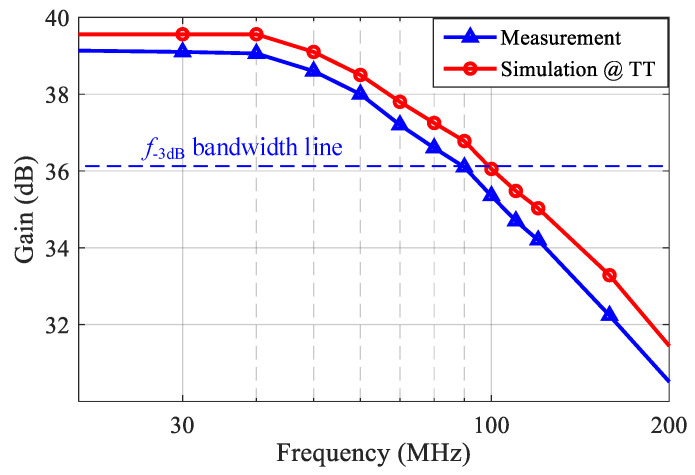
The measured frequency response of the front-end.

**Figure 19:**
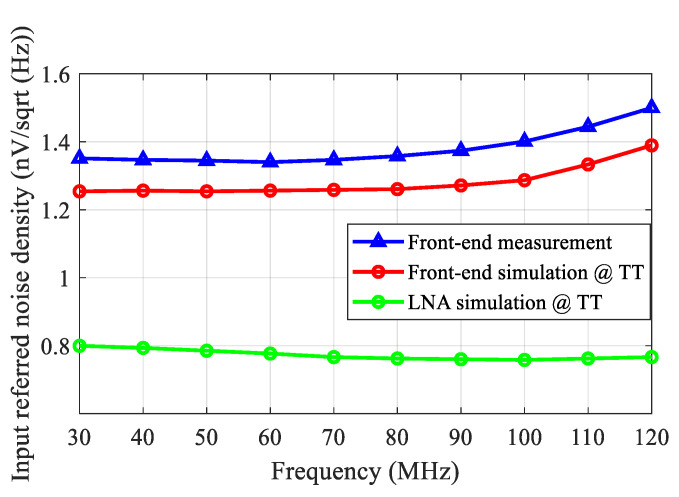
The measured input-referred noise density of the front-end.

**Figure 20:**
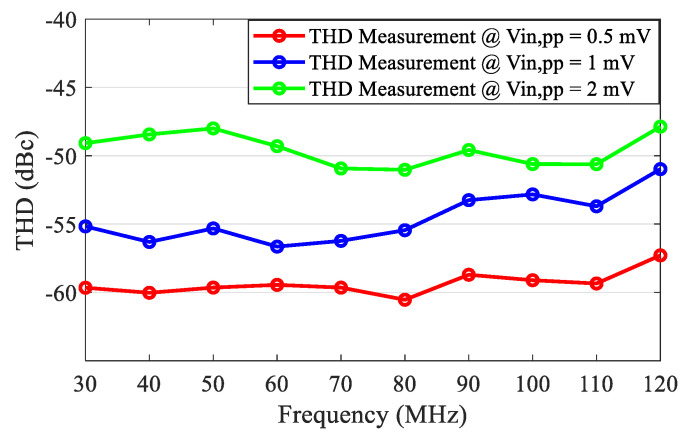
The measured THD of the front-end.

The measured S_11_ was better than −16 dB over the frequency range of 30–120 MHz, as shown in Figure 17. Setting the VGA with a 20 dB gain, the measured frequency response of the front-end is shown in Figure 18. Due to the bandwidth limitation of the VGA, the measured *f*_−3dB_ bandwidth of the front-end was 89 MHz. The noise performance of the front-end over 30–120 MHz is shown in Figure 19. The measured input-referred noise density of the front-end was 1.36 nV/sqrt (Hz) at 80 MHz, which closely matched the simulation result of 1.26 nV/sqrt (Hz). Figure 20 shows the measured THD of the front end. With V_IN, PP_ = 1 mV, the measured THD was better than −55 dBc over 30–80 MHz and was −51 dBc at 120 MHz.

The noise efficiency factor [26], which considers the overall trade-off among noise, power consumption, and bandwidth, was obtained for the proposed front end. The noise efficiency factor was defined as

(22) $${\mathrm{NEF}=V}_{\mathrm{NI},\mathrm{RMS}}\cdot \sqrt{\frac{{2I}_{TOT}}{\pi \cdot V_{T}\cdot 4kT\cdot BW}},\;$$

where $V_{\mathrm{NI},\mathrm{RMS}}$ is the total input-referred voltage noise, $I_{TOT}$ is the total current drained by the circuit, $V_{T}$ is the thermal voltage, and *BW* is the front-end bandwidth. The input-referred noise of the front-end over 30–120 MHz was 14.2 µV and the $I_{TOT}$ was 20.56 mA. The NEF of the front-end was thus determined as 2.66.

Table 2 summarizes the performance of the front-end and compares it with recently published ultrasound amplifiers. The front-end achieved a low input-referred voltage noise density, a low THD, a high *f*_−3dB_ bandwidth, and competitive power consumption, demonstrating the best NEF.

## 6. Conclusions

This paper presents a low-noise amplifier front-end for high-frequency ultrasound transducers. The LNA employs a resistive shunt-feedback configuration to simultaneously achieve a large *f*_−3dB_ bandwidth and a wideband impedance matching. To mitigate the noise in the resistive shunt-feedback amplifier, a feedforward noise-canceling technique was developed. A complementary CMOS topology was also developed to cancel the amplifier’s second-order nonlinear distortion. An ultrasound receiver front-end, including the proposed LNA and a pseudo-differential VGA, was fabricated in a standard 180 nm CMOS process. Measured at 80 MHz, the front-end achieved an input-referred noise density of 1.36 nV/sqrt (Hz), a −16.4 dB input return loss, a 37 dB voltage gain, and a −55 dBc THD, while consuming 37 mW of power. The front-end demonstrated the best NEF with a large *f*_−3dB_ bandwidth, wideband impedance matching, low noise and harmonic distortion, and competitive power consumption, making it suitable for high-frequency ultrasound transducer applications.

## Figures and Tables

**Table 1:** Simulated THD of the LNA.

P_IN_ (dBm)		−62	−56	−50
THD (dBc) @ 80 MHz	NC only (NMOS)	−55.1	−49.1	−42.7
	NC only (PMOS)	−55.7	−49.6	−43.5
	NC with complementary	−65.3	−59.3	−53.7

**Table 2:** LNA front-end performance comparison.

Parameters	This Work *	[27] *	[28] **	[29] **	[30] *	[31] *	[32] *	[33] *
Process [nm]	180	180	28	180	130	350	350	180
Power supply [V]	1.8	1.8	1.0	1.8	3	3.3	±2.5	1.8
Bandwidth [MHz]	770 ^a^/90 ^b^	100	100	30	10	75	30	33
Gain [dB]	37	17.6	20	15.2	36	20	12	19.1
Input-referred noise density [nV/sqrt (Hz)]	1.36	4.19	1.74	3.5	7.41	2.68	6.3	1.01
Total input-referred noise [µV]	14.2	−	20.8	34.9	23.4	−	35.6	5.8
THD [dBc]	−55	−	−	−	−	−	−	−53.5
S_11_ [dB]	−16	−	−	−	−	−	−	−
Power consumption [mW]	37	43	2	0.27	12.6	−	20	16.2
Noise efficiency factor	2.66	−	3.57	3.02	18.51	−	15.36	3.69
Core area [mm^2^]	0.063	0.683	0.001	−	0.22	−	−	0.363

* Measurement results; ** Simulation results. ^a^ LNA bandwidth; ^b^ Front-end bandwidth.

## Data Availability

Data sharing is not applicable to this article as authors have used publicly available references, whose details are included in the “Measurement Results and Analysis” section of this article. Please contact the authors for further requests.
